# Co-existent Paget’s Disease of the Bone, Prostate Carcinoma Skeletal Metastases and Fracture on Skeletal Scintigraphy-Lessons to be Learned

**DOI:** 10.4274/Mirt.135

**Published:** 2013-08-01

**Authors:** Luke I. Sonoda, Kottekkattu K. Balan

**Affiliations:** 1 Addenbrooke’s Hospital, Department of Nuclear Medicine, Cambridge, CB2 0QQ, United Kingdom

**Keywords:** Paget’s disease of bone, prostate cancer, scintigraphy, bone fractures, neoplasm metastases

## Abstract

Bone scintigraphy, despite being non-specific, is a very sensitive and simple investigation for patients with active Paget’s disease of the bone. Skeletal metastases and Paget’s disease may co-exist in the elderly patients as both conditions are commonly seen in this age group. Clinical and radiological correlation may help to improve the diagnostic specificity of a bone scintigram. We report a patient in whom concurrent Paget’s disease and a rib fracture became evident only on repeat scintigraphy following successful treatment of prostate carcinoma skeletal metastases.

**Conflict of interest:**None declared.

## INTRODUCTION

^99^Tc-MDP bone scintigraphy (bone scan) has been playing an important role in detecting benign and malignant skeletal abnormalities for more than five decades. Although it is well known to be sensitive in detecting osteoclastic bony lesions, it is not specific to the underlying causes of bony diseases. We present a patient in whom multiple skeletal metastases, active Paget’s disease and a rib fracture coexisted. Careful use of clinical, laboratory, radiological and scintgraphic data is important in reaching the accurate diagnosis.

## CASE REPORT

**Figure 1:**
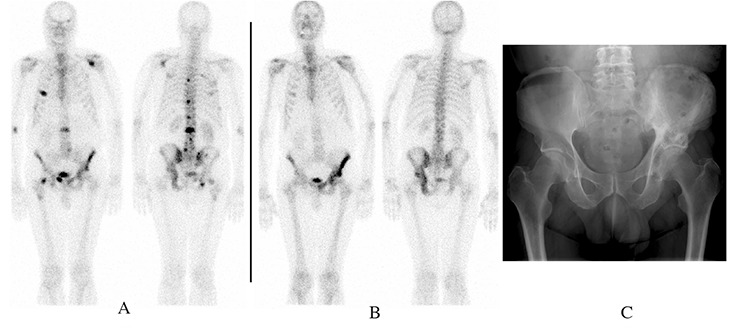
A) Whole-body bone scintigram demonstrates secondaries at multiple sites. Faint diffuse increased uptake is visible in the left hemi-pelvis B) Whole-body bone scintigram shows diffuse low-grade tracer uptake in the left hemi-pelvis suggesting active Paget’s disease. Faint focal uptake is present in the in right fourth rib (arrow)( please place the arrow). All other lesions have now disappeared. C) Radiograph of pelvis shows typical features of Paget’s disease in the left hemi-pelvis, with lytic and sclerotic cortical changes and coarse trabeculation

A 68-year old man with raised (>250ng/ml) Prostatic Specific Antigen (PSA) and biopsy-proven carcinoma of the prostate underwent hormonal treatment. A whole-body bone scintigram (Figure 1A) prior to therapy had shown multiple metastases in the vertebral column, ribs, left scapula, left fronto-orbital region, pelvis and right proximal femur. There was also faint, diffuse uptake in the left hemi-pelvis but this was deemed insufficient for a definite diagnosis of active Paget’s disease at the time. Local radiotherapy was given for back and right hip pain. Post-therapy PSA returned to normal (0.31ng/ml). A year later, he developed pain in the left hip, when the repeat bone scintigram (Figure 1B) showed total disappearance of all the previously noted lesions with the exception of persistent but faint focal uptake in right fourth rib, suggestive of a healing fracture, either due to a previous traumatic fracture or a pathological fracture due to prostatic metastases. Also, diffuse, low-grade uptake consistent with active Paget’s disease was seen in the left hemi-pelvis. The latter diagnosis was confirmed by plain radiograph (Figure 1C) and a raised serum alkaline phosphatase.

**Literature Review and Discussion**

It is not infrequent for Paget’s disease and osseous metastases to coexist in an elderly patient and this possibility has to be kept in mind in order to avoid mistaken diagnosis. Further, management of the two conditions is different. In Paget’s disease, the tracer uptake on scintigraphy is often intense, well demarcated and evenly distributed in the affected skeleton (1,2). It tends to preserve and even enhance the normal anatomic configuration of the involved bones (3,4). In contrast, metastatic disease usually presents with random spotty lesions or patchy dense tracer uptake. It tends to obliterate rather than reinforce normal bone outlines (5) If it involves two areas of the same bone, the intervening bone usually appears normal on scintigraphy unlike the homogeneous tracer uptake seen in Paget’s disease (6). There is usually no bony deformity or overgrowth. Plain radiography is extremely useful in this situation and frequently resolves the problem (7). 

In our patient, it was only after the regression of bone metastases following hormonal treatment and local radiotherapy that Paget’s disease in the left hemi-pelvis became apparent on skeletal scintigram. There have been case reports in the past of these two conditions occurring in the same patient (8,9). In the case discussed here, it is possible that early Paget’s disease was present in the left hemi-pelvis at the time of first bone scan when bone metastases were documented. The features were, however, not typical and it was not possible to diagnose the condition even with the benefit of hindsight. Only 65% of Pagetic bone shows abnormalities in both radiographs and bone scintigrams (10). In the remaining 35%, two-thirds are recognized only on bone scan alone and these are associated with early disease. One-third of the remaining lesions are appreciated only on radiographs alone and these are either sclerotic or ‘burned out’ lesions. It has been reported that lesions detected by radiograph only were always asymptomatic whereas lesions seen on bone scintigram only were symptomatic in most patients (2). This patient had painful left hip when the second bone scan and pelvic radiograph were obtained. No radiological comparison was available at the time of the first nuclear medicine study.

The persistent, albeit with reduced intensity, tracer uptake in the right fourth rib seen in our case is unlikely to be due to metastasis as all the other lesions had disappeared in response to treatment. This is further supported by the return of PSA to normal level. However, it has to be noted that it is often hard to make a clear diagnosis of a focal rib tracer uptake. The possibilities of traumatic rib fracture and pathological rib fracture due to metastases should always be kept in mind in differential diagnosis, as the duration of healing period and the tracer activity and may depend upon the severity of the fracture regardless of the cause.
